# Diffusion tractography for awake craniotomy: accuracy and factors affecting specificity

**DOI:** 10.1007/s11060-021-03795-7

**Published:** 2021-07-01

**Authors:** Natalie L. Voets, Pieter Pretorius, Martin D. Birch, Vasileios Apostolopoulos, Richard Stacey, Puneet Plaha

**Affiliations:** 1 Department of Neurosurgery, Oxford University Hospital NHS Foundation Trust, John Radcliffe Hospital, West Wing, L3, Oxford, Oxfordshire OX3 9DU UK; 2 Nuffield Department of Surgery, University of Oxford, Oxford, Oxfordshire UK; 3 Department of Neuroradiology, Oxford University Hospital NHS Foundation Trust, John Radcliffe Hospital, Oxford, Oxfordshire UK; 4 Nuffield Department of Anaesthesia, Oxford University Hospital NHS Foundation Trust, John Radcliffe Hospital, Oxford, Oxfordshire UK

**Keywords:** Glioma, Diffusion tractography, DTI, Awake surgery, Stimulation

## Abstract

**Introduction:**

Despite evidence of correspondence with intraoperative stimulation, there remains limited data on MRI diffusion tractography (DT)’s sensitivity to predict morbidity after neurosurgical oncology treatment. Our aims were: (1) evaluate DT against subcortical stimulation mapping and performance changes during and after awake neurosurgery; (2) evaluate utility of early post-operative DT to predict recovery from post-surgical deficits.

**Methods:**

We retrospectively reviewed our first 100 awake neurosurgery procedures using DT- neuronavigation. Intra-operative stimulation and performance outcomes were assessed to classify DT predictions for sensitivity and specificity calculations. Post-operative DT data, available in 51 patients, were inspected for tract damage.

**Results:**

91 adult brain tumor patients (mean 49.2 years, 43 women) underwent 100 awake surgeries with subcortical stimulation between 2014 and 2019. Sensitivity and specificity of pre-operative DT predictions were 92.2% and 69.2%, varying among tracts. Post-operative deficits occurred after 41 procedures (39%), but were prolonged (> 3 months) in only 4 patients (4%). Post-operative DT in general confirmed surgical preservation of tracts. Post-operative DT anticipated complete recovery in a patient with supplementary motor area syndrome, and indicated infarct-related damage to corticospinal fibers associated with delayed, partial recovery in a second patient.

**Conclusions:**

Pre-operative DT provided very accurate predictions of the spatial location of tracts in relation to a tumor. As expected, however, the presence of a tract did not inform its functional status, resulting in variable DT specificity among individual tracts. While prolonged deficits were rare, DT in the immediate post-operative period offered additional potential to monitor neurological deficits and anticipate recovery potential.

**Supplementary Information:**

The online version contains supplementary material available at 10.1007/s11060-021-03795-7.

## Introduction

Surgery remains the first-line treatment for intrinsic brain tumors. Surgery has undergone increasing refinement with adjuncts that help maximize tumor resection and reduce morbidity. Evidence for the added value of emerging intra-operative approaches, however, remains low [1].

Neuronavigation incorporating diffusion tractography (DT) has received particular attention for its potential to facilitate glioma surgery [2, 3]. Maximal resection optimizes overall and progression-free survival in low-grade [4, 5] and high-grade gliomas [6]. However, the often-eloquent location of gliomas [7] creates challenges in deciding how to maximize resection without causing neurological deficits. Among the primary factors limiting resection is the location of eloquent subcortical white matter pathways [8]. Electrical stimulation mapping and neurophysiological monitoring studies highlight the need to spare deep fiber tracts to maximize quality of life [9]. Pre-surgical indicators of the location of functionally relevant tracts, therefore, have great potential to assist with surgical decision-making.

MRI-based diffusion tractography (DT) is the only technique available to estimate fiber tracts in vivo. Although an indirect technique [10], the topography of tracts estimated from DT shows high correspondence with neuroanatomical dissections [11]. When applied in brain tumor patients, pre-operative DT can depict varied effects (including displacement or infiltration) of space occupying lesions on fiber tracts in individual patients [12, 13]. These applications motivate clinical interest in pre-operative DT to inform safe surgical approaches [14], anticipate the likely extent of resection [15–17] and plan the need and focus for electrical stimulation [12, 18].

Evaluations against intra-operative electrical stimulation highlight that factors including tumor-associated edema and brain shift [19] reduce the accuracy of pre-operative DT. Nevertheless, the predictive value of DT for specific fiber tracts is generally high [20] when allowing a margin of 5–7 mm, consistent with the expected electrical current spread [18, 21]. Of course, detecting the presence of tracts does not prove these are essential to function, driving the need for intra-operative tract monitoring. However, awake surgery is not feasible in all patients, and not infallible [22]. Consequently, there is growing interest in the potential of DT not only to anticipate intra-operative findings, but—crucially—to predict post-operative functional outcomes and recovery [23–26].

We analyzed findings in 100 patients at our institution who underwent awake surgery for intrinsic brain tumors using DT neuronavigation combined with subcortical stimulation. Our aims were to:

1. Confirm DT sensitivity, specificity and accuracy for 5 primary fiber tracts, evaluated not only against intra-operative stimulation but also against behavioral change during/after surgery.
2. Identify factors contributing to discrepancies between pre-surgically DT predicted tract locations and intra-operative stimulation.
3. Explore if post-operative indications of fiber tract damage inform recovery potential.

## Methods

### Patients

We reviewed all adults undergoing awake surgery for intrinsic brain tumors at our institution between May 2014 and 31 May 2019. Patients were selected for awake surgery if intraoperative assessment of speech, sensorimotor or visual functions was felt to be important to achieve maximal resection without morbidity based on the location of the tumor. The first 100 surgeries using intra-operative subcortical stimulation were analyzed. Patients were reviewed at a multidisciplinary meeting and dedicated surgical neuro-oncology clinic to assess performance status and candidacy for awake neurosurgery.

### MRI

Patients underwent MRI approximately 24 h before surgery on a 1.5 T Philips Achieva. Scans included volumetric pre- and post-Gadolinium T1-weighted scan, axial T2-weighted FLAIR and diffusion (b-value: 1200 s/mm^2^, 32 directions, 2.5 mm^3^ resolution). 98 patients underwent post-operative MRI approximately 24 h after surgery, including post-operative DT in 51 patients. Two patients had post-operative CT. Post-operative MRIs were evaluated independently by a consultant neuroradiologist to determine extent of resection.

### Fiber reconstructions

Diffusion data were analyzed using commercial software (Brainlab iPlan® (Munich, Germany) or Medtronic StealthStation® S7 (Louisville, USA) using standard processes. Tracts were selected based on tumor location and included the arcuate/superior longitudinal fasciculus (SLF), inferior fronto-occipital fasciculus (IFOF), corticospinal tract (CST), optic radiations (OR) and inferior longitudinal fasciculus (ILF). Tractography plans were exported to the neuronavigation system (see Online Resource).

### Awake neurosurgery protocol

Patients were operated according to a Monitored Anesthesia Care (MAC) or Conscious sedation protocol (Online Resource). Subcortical stimulation was performed when approaching tumor margins, or where DT indicated proximal fibers. Stimulation started around 6–8 mA, descending to a lower voltage on approaching tracts. Surgical resection alternating with stimulation proceeded until functional margins were encountered or planned tumor resection was achieved. Subcortical stimulation was not always reliable/possible due to variable compliance levels or clinical events (e.g. seizures). In some cases, resection continued without stimulation, provided behavioral monitoring remained possible; in other words, patients maintained responses to test stimuli/instructions but did not comply sufficiently for stimulation and/or further stimulation was contraindicated by intra-operative focal seizures. Performance deterioration in specific tract-related functions of sudden onset, unrelated to seizures, pain, bleeding or dynamic brain traction was recorded as behavioral change without clear stimulation-induced response.

### Intra-operative assessments

Tract functionality was assessed using standard tests [27, 28], including naming and word repetition (arcuate, SLF), Pyramids and Palm Trees semantic association test (IFOF), naming objects in opposing visual quadrants (ORs), visual naming and reading (ILF). CST proximity was assessed through involuntary movements/sensations, and loss in muscle power/range. The location of stimulation sites was visualized on the neuronavigation system and recorded.

### Peri-operative performance

Immediate post-operative neurological outcome was determined over 24–48 h of in-patient observation. Neurological status was determined using the World Health Organization (WHO) performance status, supplemented as appropriate with neuropsychological tests probing language function (fluency, repetition, naming, reading, writing). Muscle power was measured using the Medical Research Council 5-point muscle scale. After discharge, patients returned for out-patient review within 2 weeks of surgery. Clinical reviews were 3 monthly for ‘low-grade glioma’ and bi-monthly for ‘high-grade glioma’ patients until the start of adjuvant treatment.

### DT analyses

A *True positive* (TP) prediction was defined as a tract identified by DT and confirmed by intra-operative stimulation/behavioral change. DT findings were classified *True negative* (TN) if no tract was indicated near the tumor, nor found with stimulation. A *False positive* (FP) was defined as a tract identified by DT but not identified intra-operatively. Predictions were *False Negative* (FN) if no tract was located by DT but was found through stimulation. See Online Resource Fig. S1. Sensitivity was calculated as (TP)/(TP + FN); specificity as (TN)/(TN + FP) and accuracy as (TN + TP)/(TN + TP + FN + FP). Tracts were excluded from analysis if there was adjacent residual tumor on post-operative MRI or indications of atypicality (left-handedness or prior surgery combined with absence of expected symptoms intra-/post-operatively).

### Post-operative DT

Fiber tracts were separately reconstructed from the post-operative scans and visually compared to pre-operative trajectories for gross evidence of surgical tract damage. We expected deficits would persist given evidence of tract injury, or be transient if tracts remained intact.

### Statistical analyses

Statistical analyses were performed using SPSS v25. Group comparisons consisted of independent samples t-tests. Chi-square tests compared the frequency of specific DT predictions (i.e. True Positive, etc.) by performance outcome, and the contribution of histopathological diagnosis on DT predictions. Significance was set at p < 0.05.

## Results

### Demographics

Table 1 details clinical and histo-molecular data for 100 awake procedures in 91 patients (48 men, mean age: 49.2 years, range: 19–74 years). Resection was radiologically gross total (100% tumor resection, 40 patients), near total (90–99% resection, 28 patients) or subtotal (< 90%, 32 patients).

**Table 1:** Clinical and demographic data for 91 patients constituting our first 100 awake surgeries

(Parameter/Variable)	Value/Number (%)
Age (years), n = 91	
Mean	49.2
Range	19–74
Sex (n = 91)	
Male	48
Female	43
Symptoms at presentation (n = 91)	
Seizure/collapse	49
Headache/nausea/confusion	6
Neurology	25
Incidental finding	2
Surveillance	8
Unknown	1
Hemisphere of surgery (n = 100)	
Left	66
Right	34
Type of surgery (n = 100)	
First operation	79
First awake surgery (re-do operation)	12
Repeat awake surgery	9
Radiological tumor resection (n = 100)	
Gross total	40
Near total (> 90% resection)	28
Subtotal (< 90%)	32
WHO tumor grade (n = 100)	
*II*	*20*
Oligodendroglioma	11
Astrocytoma	9
*III*	*27*
Oligodendroglioma	8
Astrocytoma	18
Clear cell ependymoma	1
*IV*	*51*
*Metastasis*	2
IDH status	
IDH+	43
IDH−	43
IDH unavailable	5

IDH-mutated tumors (denoted as “IDH + ”) included the common IDH1R132H mutation as well as rarer IDH1 and IDH2 variants

*WHO* World Health Organization, *IDH* Isocitrate dehydrogenase

### Fiber tract evaluations

**Fig. 1:**
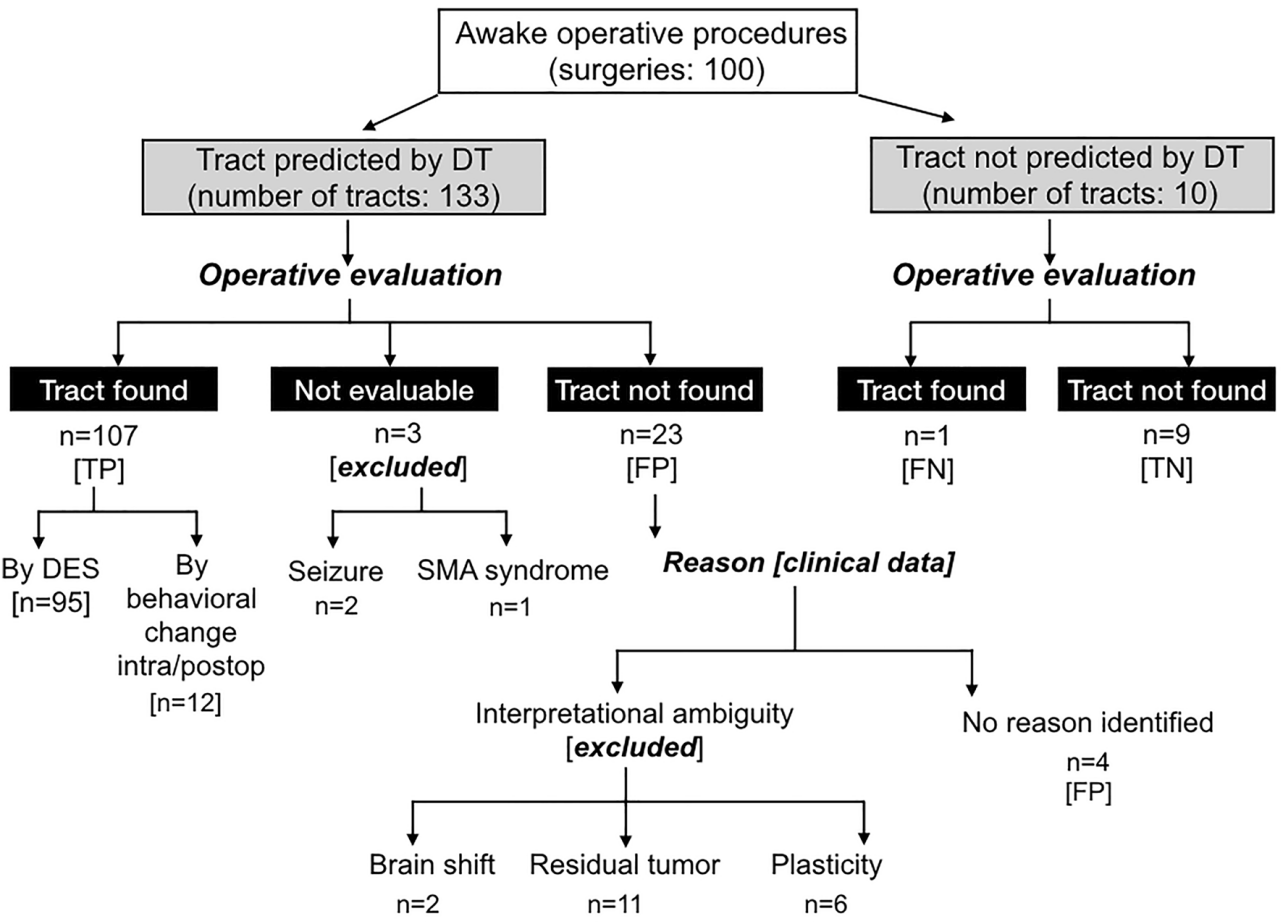
Pre-operative fiber tract predictions evaluated against intra-operative subcortical stimulation and post-operative outcomes. Diffusion tractography (DT) predicted fiber locations evaluated against intra-operative direct electrical stimulation (DES) and then refined by clinical data including performance outcomes and resection extent. *TP* true positive, *FN* false negative, *TN* true negative, *FP* false positive

143 tracts were evaluated during 100 surgeries (Fig. 1). 51 were CST, 29 arcuate/SLF, 37 IFOF, 19 optic radiations and 7 ILF.

### DT true positive

95 tracts were identified with subcortical stimulation. Another 12 were identified through intra-operative performance decline (n = 5), post-operative deficits (n = 4) or both (n = 3), each involving language tracts (4 Arcuate, 7 IFOF, 1 ILF). Together, 107 DT predictions were classified ‘TP’.

### DT true negative

Stimulation was performed to verify 9 tracts which DT indicated should be remote from the surgical margins. For all 9, no stimulation-related behavioral changes occurred, confirming ‘TN’ predictions.

### DT false negatives

**Fig. 2:**
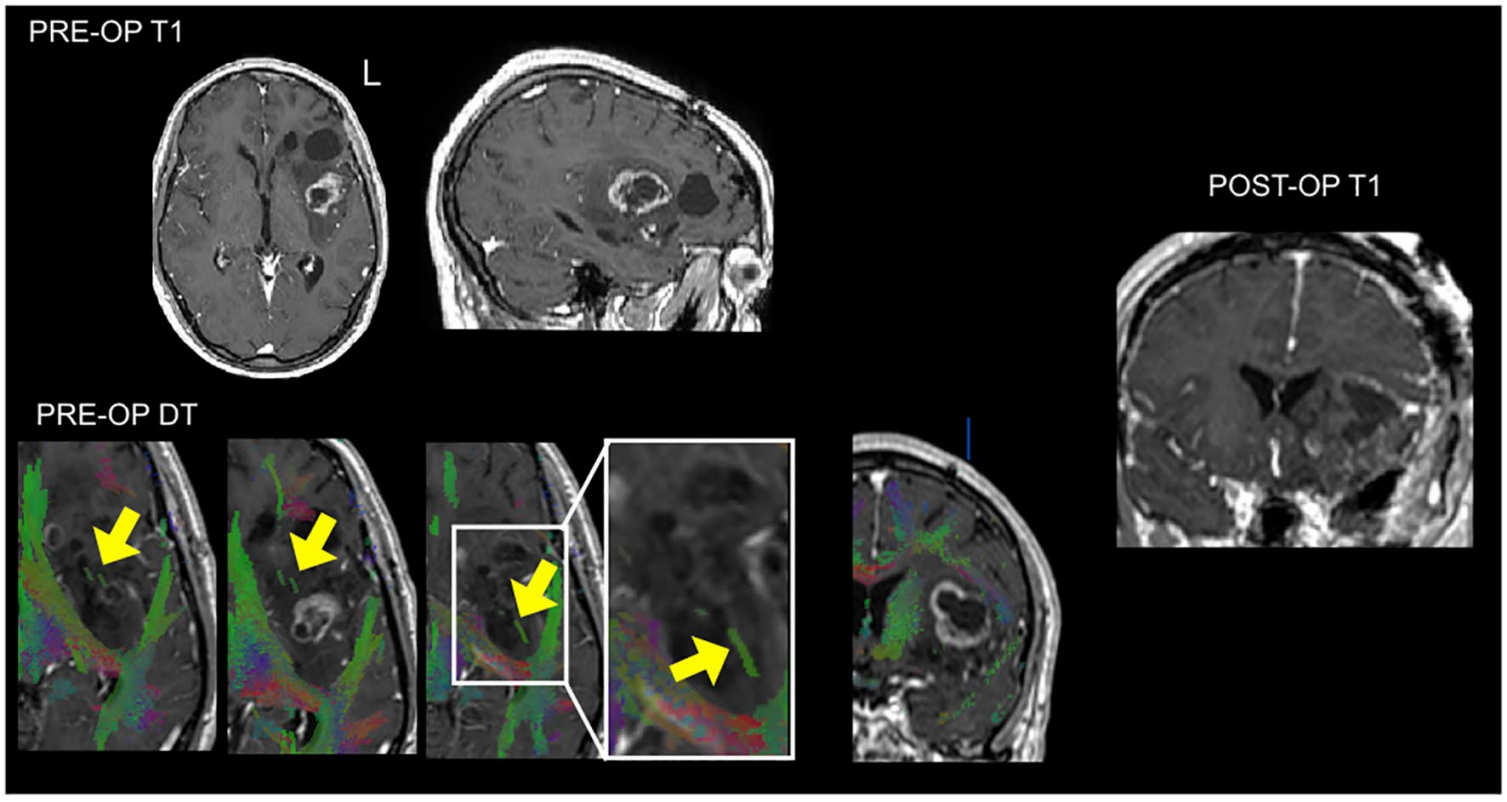
Case with discordance between pre-operative DT and intraoperative stimulation. A 41-year-old right handed man on imaging surveillance for a known IDH mutated astrocytoma. Although an island of white matter and sparse ‘tracts’ were visible within this heterogeneous transforming lesion, pre-operative diffusion tractography (DT) did not convincingly identify the inferior fronto-occipital fasciculus running through the tumor, even at a reduced fractional anisotropy tracking threshold (0.17). However, subcortical stimulation during awake surgery elicited semantic paraphasias and naming errors at this location, indicating an eloquent IFOF. The patient experienced transient slight language deterioration affecting naming and repetition

One tract was not accurately predicted by DT. This patient showed malignant transformation of a known diffuse left temporo-fronto-insula astrocytoma. DT indicated white matter within the tumor. However, the IFOF was not reliably reconstructed due to confounding pathological signal in the temporo-parietal white matter (Fig. 2). Intra-operative stimulation induced semantic paraphasias and naming errors, indicating eloquent IFOF within the tumor. The patient experienced mild, transient post-operative language deficits. This result was therefore a ‘FN’.

### DT false positives

Four stimulation-silent but DT-predicted tracts (2 optic, 1 ILF, 1 CST, 3 patients) were not explained by identified clinical factors and classified ‘FP’ predictions. None experienced deficits.

### Exclusions

Stimulation induced seizures in two patients. Both recovered but had prolonged post-ictal states, limiting further stimulation mapping. One further patient developed an intra-operative supplementary motor area syndrome, precluding evaluation of the DT-predicted CST.

19 additional DT-predicted tracts were not identified intra-operatively. Post-operative MRI revealed residual tumor adjacent to 11/19. For 6 others, the absence of stimulation-induced errors indicated atypical functional organization. Three ‘silent’ tracts (1 IFOF, 1 ILF, 1 arcuate) occurred in two left-handed patients operated for a newly diagnosed left hemisphere glioma. Neither patient had language symptoms before, during or after surgery, indicating right hemisphere dominance for language. The remaining three stimulation-silent tracts (1 arcuate, 1 CST and 1 IFOF) occurred in three patients re-operated for tumor recurrence at 8, 3.7 and 3.4 years after initial surgery following full (n = 2) or partial (n = 1) behavioral recovery. Finally, substantial brain shift occurred in two glioblastomas. The pre-operative fiber tract locations (optic radiations in both) displayed on neuronavigation and the intra-operative location of stimulation visualized through the microscope became misaligned during surgery. The stimulation sites remained remote to predicted tracts, and neither patient experienced deficits. Since the DT predictions could not be conclusively verified these 22 tracts were omitted from sensitivity/specificity analyses.

### Tumor grade

The frequency of DT tract predictions (TP, etc.) did not differ by tumor grade ($\chi$(6) = 4.3, p = 0.64), or between ‘low’ versus ‘high’ grade tumors ($\chi$(3) = 4.25, p = 0.11).

### Post-operative deficits

Notwithstanding minor wound leaks/infections (n = 4), surgical complication rates were low; 41/100 awake surgeries (41%) resulted in a post-operative performance decline, relating to 52 fiber tracts (Online Resource Fig. S2). Most declines were transient. Nine patients had persisting deficits (lasting > 3 months) but not all resulted from surgery: four arose from tumor progression and one patient deteriorated upon starting radiotherapy. Therefore, definite prolonged deficits occurred after four surgeries (4%), each involving sensorimotor functions. Tumor histological grade did not differ between patients with or without post-operative deficits (t = 1.63, p = 0.11).

### DT sensitivity, specificity, accuracy

107 tracts were classified TP, 1 as FN, 9 TN, and 4 FP (Fig. 2), yielding sensitivity of 92.2%, specificity of 69.2% and accuracy 95.9%. For values per fiber tract, see Table 2.

**Table 2:** Sensitivity and specificity of DT predictions according to fiber tract

Tract	TP	TN	FP	FN	Exclusions	Sensitivity (%)	Specificity	Accuracy (%)	EARLY DEFICIT^a^
CST (n = 51)	32	6	1	0	11	84.2	85.7%	97.4	N = 20
Arcuate **(n = 29)**	24	2	0	0	4	92.3	100%	100	N = 13
IFOF **(n = 37)**	33	1	0	1	2	97.1	100%	97.1	N = 13
OR **(n = 19)**	13	0	2	0	4	100	N/A*	86.7	N = 4
ILF **(n = 7)**	5	0	1	0	1	100	N/A*	83.3	N = 2

Sensitivity, specificity and accuracy of diffusion tractography (DT) predictions for individual fiber tracts

*CST* corticospinal tract, *IFOF* inferior fronto-occipital fasciculus, *OR* optic radiations, *ILF* inferior longitudinal fasciculus, *TP* True Positive, *TN* True Negative, *FP* False Positive, *FN* False Negative, *N*/*A* Not available

*In the absence of True Negative instances, specificity cannot be meaningfully quantified

^a^Early deficit = new or worse deficit emerging during surgery or in the immediate post-surgical period. Almost all deficits were transient, with the very small number (4%) of persisting deficits each involving sensorimotor functions (CST), see “Results” section

### Post-operative tract evaluations

**Fig. 3:**
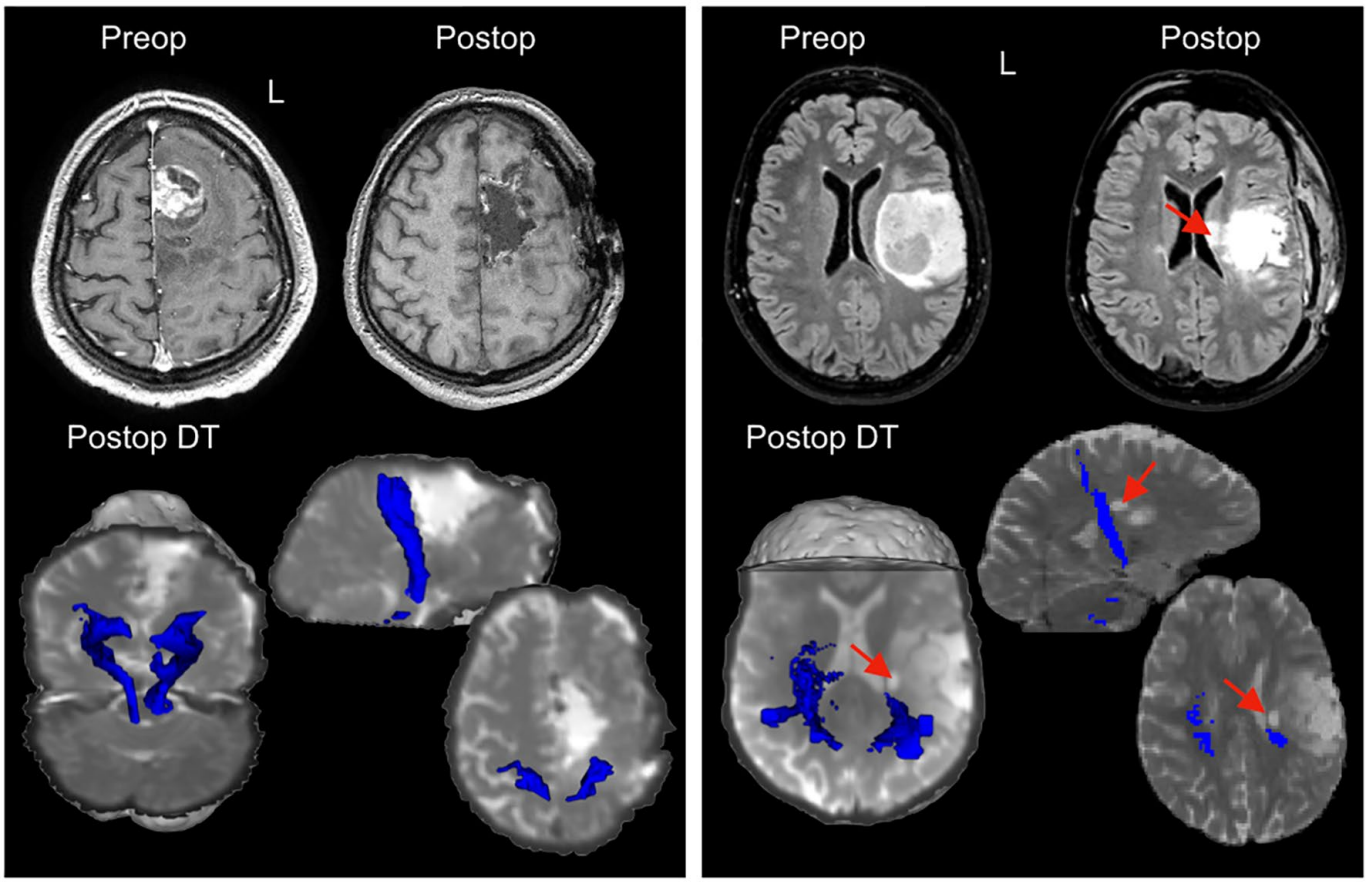
Post-operative diffusion tractography to inform recovery potential in two patients with post-operative deficits. Left panel: A 42-year-old right handed male developed speech and motor deficits during resection of a radiologically transforming glioma in the medial frontal lobe. Speech recovered quickly over the first 3 days following surgery. Right hand function was absent immediately following surgery (0/5, MRC scale) and right leg function was significantly impaired (2/5). Post-operative diffusion tractography (DT) acquired 24 h after surgery showed preserved corticospinal (CST) fibers, supporting the diagnosis of a supplementary motor syndrome likely to recover. Hand function gradually returned on the 6th post-operative day; leg strength returned to normal by 1 month. Right panel: A 34-year-old right handed female developed sudden limb weakness. Immediate post-operative MRI identified an area of infarct affecting the corona radiata and body of the caudate nucleus. Post-operative DT demonstrated an asymmetrical CST, with fewer detected tracts adjacent to the infarct. Residual connections reaching the primary motor cortex, however, suggested some recovery potential. Hand function improved over the first 10 days after surgery, with additional gains in lower limb function with therapy which continued for 3 months post-surgery. However, she was still occasionally using a stick to walk

Post-operative DT was available in 51 patients, including 20 of those who experienced post-surgical performance declines. In 14/20 (70%), deficits recovered rapidly. Three patients had early tumor progression contributing to neurological decline. The remaining three patients were among the four reported above with de novo deficits persisting beyond 3 months: one experienced sensory symptoms without loss of mobility, one had persisting motor SMA syndrome, and one had residual lower limb weakness. Post-operative DT showed intact CST fibers in the patients with sensory symptoms and motor SMA syndrome. In contrast, the patient with persisting limb weakness had a small infarct involving the corona radiata, causing a post-operatively asymmetric, ipsilaterally reduced CST (Fig. 3).

## Discussion

We confirmed pre-operative DT as a reliable tool for neuronavigation in neuro-oncology surgery. Addressing our first study aim, across 100 awake surgeries, DT offered 92.2% sensitivity and 95.9% accuracy to localize tracts. Secondly, lower tract-average specificity (69.2%) reflected inherent differences between *localizing* fibers versus establishing their *functionality*. DT predicted the location of some tracts not evaluable through stimulation. Therefore, stimulation offers an important but incomplete validation of DT and maximal benefits may arise from using both techniques together. Finally, early post-operative DT offered added potential to anticipate recovery in patients with performance declines.

### Accuracy of pre-operative DT

Our first study aim was to determine the sensitivity, specificity and accuracy of DTI in our sample of patients operated awake with subcortical stimulation. While numerous studies report using pre-operative DT for neuronavigation, few have quantified its sensitivity. Bello and colleagues [21] reported high sensitivity (88.6–98.2%) and specificity (100%) of DT predictions for motor and language pathways when assessed against intra-operative stimulation. Zhu et al. [29] obtained similar values (92.6% sensitivity, 93.2% specificity) for CST DT. We recorded similar sensitivity (84.2–100%) and specificity (85.7–100%) for 5 established surgically-relevant fiber tracts. We used more comprehensive criteria to evaluate DT, since ‘gold standard’ intra-operative subcortical stimulation varies in reliability and post-operative deficits can still arise [22]. When resecting tumor near eloquent tracts, some performance declines occurred without consistent stimulation findings (12 tracts, 9%). Behavioral changes offer important additional validation of DT, since their presence indicates proximity to eloquent tracts DT aims to help preserve. We analyzed a large sample of patients operated awake, offering a way to directly observe patient behavior to assess tract proximity. However, many glioma surgeries are performed under general anesthesia. Motor/sensory evoked potentials offer an alternative method to minimize deficits when operating near sensory pathways with the patient awake or asleep [30].

### When results diverged

Our second aim was to identify factors contributing to discrepancies between pre-operative DT and intra-operative stimulation. These included:

#### Stimulation identifying tracts not seen with DT

DT failures were rare, affecting 1/143 tracts (< 1%). Insula gliomas can destroy the IFOF, and absence of IFOF in DT is predictive of achieving > 80% resection [17]. Conversely, in our case, DT did not identify one eloquent IFOF. This case highlights known challenges for DT when pathological signal (e.g. infiltrating tumor/edema) affects critical white matter regions, such as the temporo-parietal junction where numerous tracts intersect [31]. Diffusion acquisition sequences with high spatial ($\le$ 2 mm isotropic voxels) and angular ($\ge$ 60 gradient directions) resolution, analyzed using sophisticated research models, improves DT in these regions [32]. Nonetheless, the potential for failures when diffusivity is disrupted re-affirms the need for confirmatory intra-operative monitoring whenever possible.

#### Stimulation not identifying DT-predicted tracts

Three main factors explained why subcortical stimulation did not elicit behavioral change for 22 DT-predicted tracts:

1. Brain shift has been extensively described and affects neuronavigation based on pre-operative imaging. Excessive brain shift affected 2 (1%) of our tract evaluations. Intraoperative MRI [33] or more accessible navigated ultrasound offer potential for real-time DT ‘correction’, but await further validation.
2. Residual tumor surrounded 11 DT-predicted tracts (8%). In these cases, stimulation likely did not reach the targeted tract.
3. Functional variability. The arcuate, IFOF and CST generally produce consistent behavioral changes. Others, such as the ILF [34], appear functionally heterogeneous. Some studies omitted specificity analyses for functionally variable tracts [21]. Patients may also show atypical functional organization due to left-handedness, slow-growing pathology or previous surgery (reviewed in [35]). Absence of stimulation-induced errors in these scenarios does not reflect inaccuracies of DT, but instead emphasizes differences in each tool’s purpose. DT predicts the *location* of tracts, making no assumption about function, while stimulation evaluates tissue *functionality* without knowledge of tract locations. Which tract was stimulated is inferred from a patient’s response [9]. However, some results are ambiguous (e.g. several tracts, when stimulated, induce anomia) or inconsistent (based on stimulation angle and current spread). 12 tracts were identified based on characteristic symptoms that could not be repeatedly, non-consecutively localized with stimulation, reflecting susceptibility to small variations in application. DT and stimulation are, therefore, highly complementary. Evaluation of one against the other neglects valuable independent information each technique provides.

Only four stimulation-silent DT-predicted tracts remained unexplained. In one left temporal glioblastoma patient, stimulation did not locate the ILF or optic radiations. Since the predicted IFOF, situated lateral to the optic radiations, was confirmed, optic fibers were likely not stimulated. Additionally, no visual symptoms were elicited through stimulation in a patient with a right medial parietal glioblastoma and near total resection. We did not conduct post-operative ophthalmological assessment and therefore cannot rule out subtle visual field deficits. Finally, the DT-predicted CST was not encountered in one patient with a left parietal glioblastoma who had gross total resection. Pre-operative DT indicated peri-tumoral edema displacing the CST. Due to an intra-operative cardiac event, resection stopped once visible tumor had been removed without attempting to reach functional boundaries. Classification of all four tracts as FP is therefore conservative, since resection likely did not reach at least two (excluding which, specificity becomes 81.8%).

### Contributions to glioma surgery

Benefits of pre-operative DT in planning and performing glioma resections were reviewed recently [36, 37]. DT is in our experience most helpful for a patient-centric approach and intra-operative guidance. Awake craniotomy is offered when an intrinsic tumor is located adjacent to presumed eloquent tissue, since striking a functional-oncological balance is vital [38]. Patients have a better understanding of surgical risks, a key part of the consent process, when the relationship between their tumor and surrounding fibers is discussed with them. Secondly, anticipating through DT the location of tracts helps the surgeon both to plan the surgical approach [12, 17, 39] and where to target subcortical stimulation [21], especially when the length of surgery may not permit continued stimulation, or in areas (e.g. temporal fossa floor/falx) where patient discomfort can arise. Finally, DT-neuronavigation offers the surgeon confidence in proceeding with conservative resection when clinical factors preclude awake mapping, as in 3 (3%) of our patients. At least for sensory pathways, DT can also complement neurophysiological monitoring techniques (e.g. motor and/or sensory evoked potentials) that are well-established to help achieve maximal resections and reduce new motor deficits when operating on patients either awake or asleep [40–42]. Additional DT applications include minimizing surgical morbidity and predicting extent of resection. A randomized controlled trial reported fewer performance deteriorations after surgeries informed by DT than performed without DT [26]. Concordantly, a recent prospective study reported positive predictive value of DT for resection extent [43], confirming previous findings [17].

The final aim of our study was to explore the potential for *post-operative* DT to predict functional recovery. Recovery from surgical white matter damage is thought to be limited [8]. While post-operative complications and brain swelling contribute to *transient* deterioration after surgery, the main concern is to rule out damage to eloquent tracts producing *lasting* impairment. Very few (4%) of our patients experienced prolonged deficits. Nonetheless, post-operative DT distinguished a patient with a preserved CST who made a full recovery from another patient with a small infarct who required lengthy rehabilitation. Post-operative DT has rarely been reported, but another study demonstrated that post-operative language tract DT informed speech outcomes [24]. Together, these data suggest postoperative DT holds promise for predicting recovery potential.

## Limitations

Different tractography techniques produce varied results [44]. Deterministic algorithms have well-known limitations [45]. Advanced reconstruction techniques generally out-perform deterministic methods [46–49], but are not clinically licensed. Our deterministic-based results therefore under-estimate DT’s potential. Additionally, some behavioral changes result from fluctuating patient co-operation, rather than proximity to eloquent tracts. Subjectivity was present in classifying intra-operative behavioral fluctuations, but minimized through consistent evaluation by the same assessor and joint decision-making with the operating surgeon. Likewise, there is not currently any definitive method of confirming that a particular (non-sensory) behavior was absolutely the result of stimulating a given white matter pathway. Within the limits of what is known of the function and organization of white matter, our interpretations of which tracts were associated with a given stimulation error/behavioral change were guided by current practices [9] and recently proposed standardized testing protocols [27]. Finally, this was a retrospective study. Visibility of tractography results during navigation may bias the surgeon’s interpretation. Here, tractography’s accuracy was not recorded at the time of surgery, but analyzed later retrospectively and independently of the surgeon. Nonetheless, prospective randomized controlled studies are needed and underway in the UK (“FUTURE-GB”, NIHR 127930) to evaluate DT’s value in maximizing performance outcomes.

## Supplementary Information

Below is the link to the electronic supplementary material.

Supplementary file1 (DOCX 662 KB)
