# Supplementary material for: Diffusion tractography for awake craniotomy: accuracy and factors affecting specificity
Source: J Neurooncol. 2021 Jul 1;153(3):547–57. doi: 10.1007/s11060-021-03795-7 (PMC8280000; doi:10.1007/s11060-021-03795-7)
Supplement: Supplementary file 1 — Supplementary file1 (DOCX 662 KB) [file 11060_2021_3795_MOESM1_ESM.docx]

**Supplementary materials**

*Awake neurosurgery protocol*

All patients were operated according to a Monitored Anesthesia Care (MAC) or Conscious sedation protocol, as reported previously [1]. After positioning on the operating table, the patient’s head was secured with Mayfield head clamps (Integra life Sciences, USA) after infiltrating the pin site with local anesthetic (0.25% Bupivacaine with 1:200,000 adrenaline). Co-registering the patient’s head to the neuronavigation system, the site and size of the craniotomy were planned based on the location and access to the tumor. The scalp flap was anesthetized with a circumferential ring block (30-40ml of 0.25% Bupivacaine with 1:200,000 adrenaline) along the planned incision and the base of the scalp flap. Sedation was controlled using intravenous infusions of Propofol and Remifentanil with continuous titration, adjusted to the stage of surgery. Sedation was gradually reduced once the dura was exposed.

Resection was performed using a Sonopet® Ultrasonic Aspirator (Stryker®, MI, USA) under white light for patients with a presumed ‘low grade’ glioma. When high grade glioma was radiologically indicated, 5-Amino Levulinic acid (5-ALA) was administered 1 hour prior to surgery. In these cases, resection proceeded by alternating white and ultraviolet light using a Zeiss microscope (Carl Zeiss Microscopy GmbH, Jena, Germany) fitted with a fluorescent 400nm ultraviolet light and filters.

Once resection was complete, the patient was re-sedated while hemostasis was achieved. Dural closure was performed and the bone flap re-affixed with titanium skull plates. A subgaleal drain was inserted in some cases, depending on the size of the scalp flap, and the scalp closed with skin staples.

*DT fiber reconstructions*

Diffusion data were analyzed using commercially available neuronavigation software (Brainlab iPlan® (Munich, Germany) or Medtronic StealthStation® S7 (Louisville, USA). Brainlab pre-processing steps included eddy current correction and fitting a diffusion tensor model. After linear registration to the anatomical scan, deterministic tractography was performed using Fiber Assignment by Continuous Tracking (FACT) [2]. A region of interest was defined to display all tracts near the tumor. Medtronic regions of interest were defined on the directionally encoded color map to track individual fiber bundles. Tracts were inspected and if necessary, corrected. Tractography was performed using the default fractional anisotropy (FA) threshold of 0.2 and, in cases with extensive peri-tumoral edema, re-evaluated at a lower threshold ($\geq$0.15). Tracts were identified using a one or two-region of interest approach according to the tract, and based on a combination of anatomical knowledge and the criteria proposed by Catani & Thiebaut de Schotten [3]. Fibers of the arcuate/superior longitudinal fasciculus (SLF) are typically best recognized on a sagittal slice based on the orientation of these fibers in the fronto-temporo-parietal white matter; optionally when pathology affected this region, a second region of interest placed near pars opercularis could help identify these fibers. The inferior fronto-occipital fasciculus (IFOF) was commonly identified using a single ROI approach, depending on location of the tumor and any concomitant pathological signal change, using a mask drawn to encompass on an axial slice the white matter of the anterior limb of the external capsule, staying superiorly in the temporal stem so as to avoid the uncinate. If necessary, an alternative ROI was placed on a coronal slice at the level of the calcarine fissure encompassing the white matter laterally to identify all possible streamlines and edited to restrict the resulting fibers to those passing through the superior temporal stem/external capsule. Reconstruction of the corticospinal tract (CST) was more variable, depending on pathological effects. Typically, a 2 ROI approach was favored, starting with a large mask drawn on an axial slice to encompass the precentral gyrus where visible. A second ROI was then placed at the level of the posterior internal capsule covering infero-superiorly oriented tracts identified by their blue color on the color-encoded FA maps. When either of these 2 ROIs was not reliably identifiable due to pathological signal, alternative ROI placements combining the anterior brainstem with the precentral gyrus or internal capsule (whichever was localizable) were attempted. To identify the ILF, a 2 ROI approach was favored, identifying the antero-posteriorly oriented fibers at the level of the temporal pole (through their green appearance on the color-encoded FA map), with a second ROI placed as described above to encompass the white matter lateral to the calcarine fissure. As for the IFOF, if tumor in the temporal pole confounded tracking from this preferential mask, streamlines starting in the occipital seed (white matter adjacent to calcarine fissure) were reconstructed instead for indications of tracts coursing through the tumor or in a displaced location towards the inferior anterior temporal cortex. Tracking of the optic radiations focused on identifying the central and posterior bundles of the optic radiations using a 2-ROI approach between a first mask incorporating the lateral geniculate nucleus and the white matter lateral to it (where the lateral geniculate nucleus is usually identified by a sharp transition from green to red orientation in the color-coded FA map) and a second larger ROI covering the inferior and superior lips of the calcarine sulcus as well as the white matter lateral to it. Tracts were visually inspected and manually edited when necessary.

*Supplemental Figures*

**Fig. S1. Example pre-surgical diffusion tractography predictions compared to subcortical stimulation
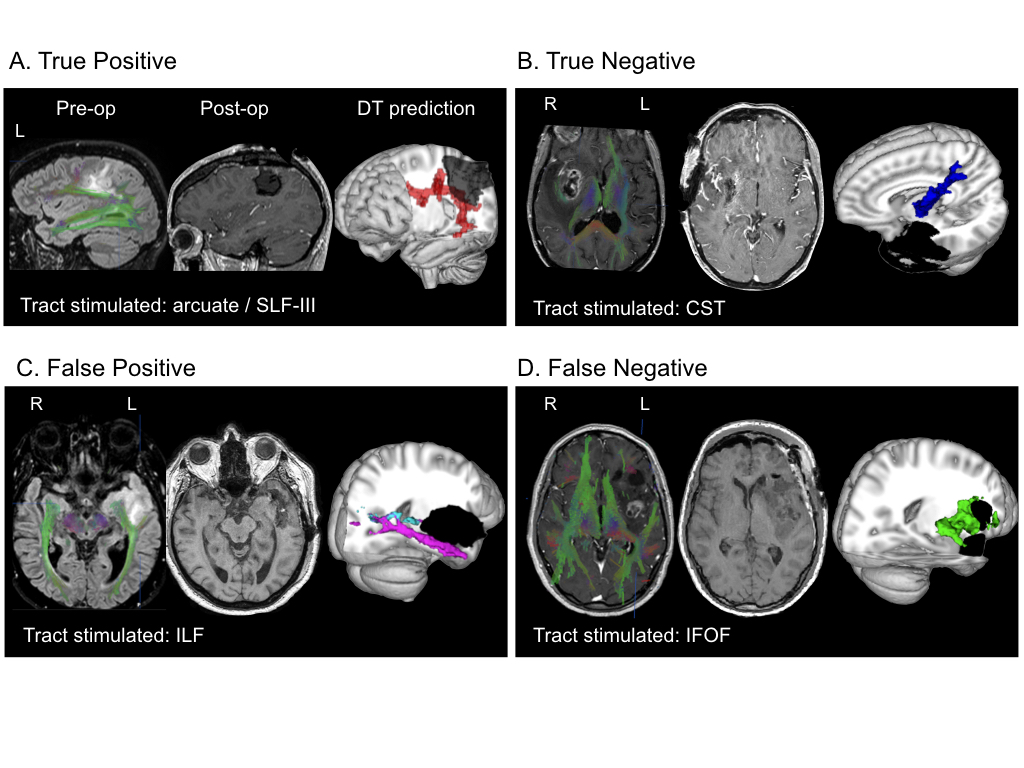
**

*Legend*. Example cases, illustrating the 4 types of pre-operative diffusion tractography (DT) predictions: A. True Positive (i.e. predicted tract, confirmed by subcortical stimulation); B. True Negative (i.e. predicted absence of proximal tract, confirmed by subcortical stimulation); C. False Positive (tract demonstrated on DT but not found with subcortical stimulation); D. False Negative (eloquent tract identified by subcortical stimulation but not reliably reconstructed with DT). SLF = Superior longitudinal fasciculus. CST = corticospinal tract. ILF = Inferior longitudinal fasciculus. IFOF = Inferior fronto-occipital fasciculus.

**Fig. S2 Post-operative deficits among 100 awake surgeries, identified causes and post-operative DT findings.**

**
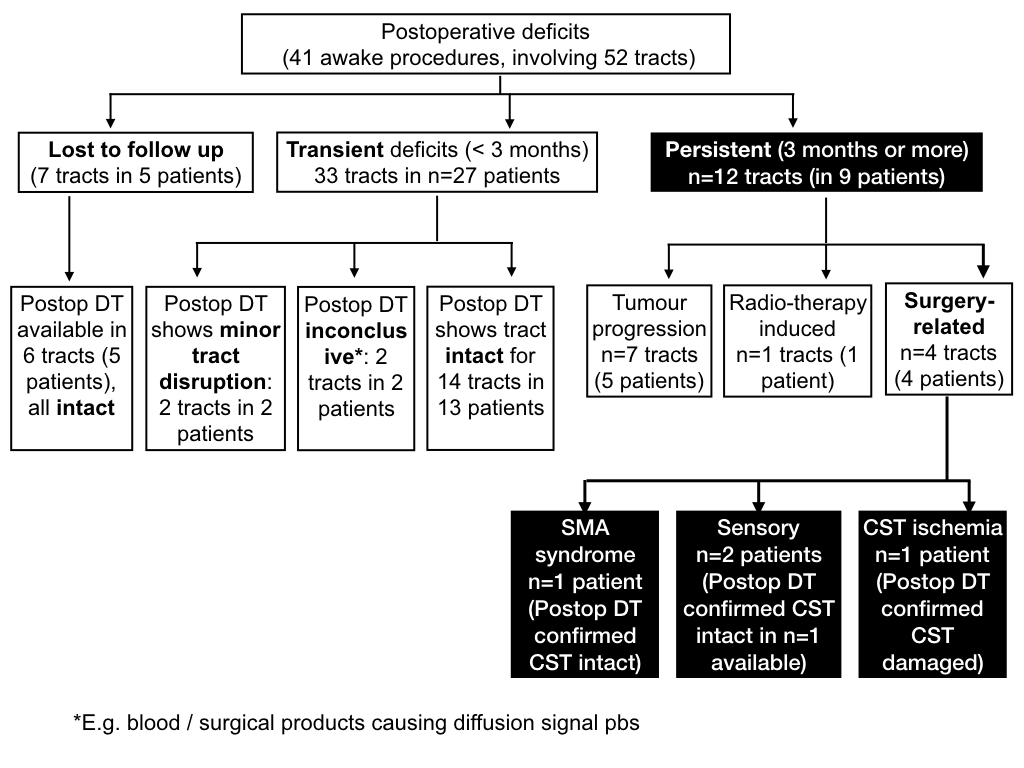
**

*Legend.* Post-operative performance deteriorations after 100 awake surgeries and their clinically identified contributing factors. Post-operative diffusion tractography (DT) was available for a subset of 51 of the 100 cases. SMA = Supplementary Motor Area. CST = corticospinal tract.

**References**

1. Ma R, Livermore LJ, Plaha P (2016) Fast Track Recovery Programme in Neurosurgery following Endoscopic and awake Intraparenchymal Brain Tumor Surgery. World neurosurgery doi:10.1016/j.wneu.2016.06.015

2. Mori S, van Zijl PC (2002) Fiber tracking: principles and strategies - a technical review. NMR in biomedicine 15: 468-480 doi:10.1002/nbm.781

3. Catani M, Thiebaut de Schotten M (2008) A diffusion tensor imaging tractography atlas for virtual in vivo dissections. Cortex; a journal devoted to the study of the nervous system and behavior 44: 1105-1132 doi:10.1016/j.cortex.2008.05.004
